# Neurotensin and its receptors in the control of glucose homeostasis

**DOI:** 10.3389/fendo.2012.00143

**Published:** 2012-11-26

**Authors:** Jean Mazella, Sophie Béraud-Dufour, Christelle Devader, Fabienne Massa, Thierry Coppola

**Affiliations:** Institut de Pharmacologie Moléculaire et Cellulaire, UMR 7275, Centre National de la Recherche Scientifique, Université de Nice-Sophia AntipolisValbonne, France

**Keywords:** neurotensin, G protein-coupled receptor, pancreas, beta cell, sortilin

## Abstract

The pharmacological roles of the neuropeptide neurotensin through its three known receptors are various and complex. Neurotensin is involved in several important biological functions including analgesia and hypothermia in the central nervous system and also food intake and glucose homeostasis in the periphery. This review focuses on recent works dealing with molecular mechanisms regulating blood glucose level and insulin secretion upon neurotensin action. Investigations on crucial cellular components involved in the protective effect of the peptide on beta cells are also detailed. The role of xenin, a neurotensin-related peptide, on the regulation of insulin release by glucose-dependent insulinotropic polypeptide is summarized. The last section comments on the future research areas which should be developed to address the function of new effectors of the neurotensinergic system in the endocrine pancreas.

## INTRODUCTION

The endogenous peptide neurotensin (NT) was discovered in 1973 in bovine hypothalami by Carraway and Leeman (1973) for its ability to induce vasodilatation. This property added to its cerebral expression indicated that the peptide could fulfill a dual function, as a neurotransmitter/neuromodulator in the central nervous system, and as a hormone in the periphery. NT is expressed in the central nervous system where it is located in neuronal synaptic vesicles (Uhl and Snyder, 1976; Bayer et al., 1991) and in the gastrointestinal tract (Goedert and Emson, 1983) in neuroendocrine cells (Atoji et al., 1995). NT is synthesized from a precursor following excision by prohormone convertases (Kitabgi, 2006).

The pharmacological and physiological effects of NT are triggered following its interaction, depending on the tissue or the cell type, by three known receptors (NTSRs). Two of them, NTSR1 and NTSR2, are classical neuropeptide receptors coupled to G-proteins (GPCR) bearing seven transmembrane domains (Vincent et al., 1999; Myers et al., 2009). The third one, NTSR3 also called sortilin (Petersen et al., 1997; Mazella et al., 1998), is a type I receptor with a single transmembrane domain, non-coupled to G-proteins, which belongs to the Vps10p containing domain receptor family (Mazella, 2001; Hermey, 2009). The heterogeneity in the structure of NTSRs, added to their ability to form homo and/or heterodimers (Martin et al., 2002; Beraud-Dufour et al., 2009; Hwang et al., 2010), underlines the complexity to study the neurotensinergic system both in the brain and in peripheral tissues.

In the central nervous system, the effects of NT include the interaction of the peptide with the dopaminergic system (Studler et al., 1988) and its ability to induce opioid-independent analgesia (Dubuc et al., 1999) and hypothermia (Popp et al., 2007). The latter property could allow the use of bioavailable NT analogs to reduce the risk of brain damage following hypoxia (Choi et al., 2012). The anti-psychotic and hypothermia effects of NT are mediated through NTSR1 (Dobner, 2005; Hadden et al., 2005; Choi et al., 2012), whereas NT-induced analgesia involves both NTSR1 and NTSR2 (Dubuc et al., 1999; Smith et al., 2012). NTSR3/sortilin is responsible for the migration and the release of cytokines and chemokines from microglial cells induced by NT (Martin et al., 2003; Dicou et al., 2004).

At the neuroendocrine point of view, the actions of NT as a regulator of anterior pituitary secretions and food intake has been recently well reviewed (Rostene and Alexander, 1997; Stolakis et al., 2010; Kalafatakis and Triantafyllou, 2011). For this reason, these sections will be only summarized at the beginning of this review focusing essentially on the role of NT in the control of glucose homeostasis. The roles of NT in insulin regulation and pancreatic cell growth have been initiated by several works (Kaneto et al., 1978; Dolais-Kitabgi et al., 1979; Wood et al., 1988). However, molecular characterization of the signaling pathways and the receptors involved in both pancreatic cell proliferation and beta cell secretion have been only recently investigated (Guha et al., 2003; Friess et al., 2003; Kisfalvi et al., 2005; Elghazi et al., 2006; Beraud-Dufour et al., 2010).

In addition, we will present the role of a NT-related peptide, named xenin, in the regulation of glucose homeostasis. Xenin is a peptide of 25 amino acids discovered in 1992 (Feurle et al., 1992), and which is synthesized from a precursor of 35 amino acids (Hamscher et al., 1996). Intriguingly, the sequence of this precursor is 100% identical to the N-terminal sequence of the mammalian coat protein α (α-COP; Chow and Quek, 1996), a cytoplasmic protein which cannot be released.

## NT AND METABOLISM

The energetic balance is regulated by the control of satiety, a domain which has evoluted following the discovery of leptin. This peptide is secreted by adipocytes and acts as a hormone directly on specific hypothalamic areas involved in the control of food intake (Jequier, 2002). Interestingly, several works reported that the effects of leptin are controlled by NT expressing neurons (Cui et al., 2006). Indeed, the anorectic effect of leptin is impaired in NTSR1-deficient mice (Kim et al., 2008), suggesting that the complex NT-NTSR1 is crucial for the action of leptin. Moreover, Leinninger et al. (2011) published an elegant demonstration showing that the action of leptin via NT neurons controls orexin release, the mesolimbic dopamine system and energy balance. In conclusion, the control of food intake is mediated by leptin on NT expressing neurons through NTSR1.

The other important aspect of the neurotensinergic system in the periphery concerns the control of nutrient absorption. Rapidly following its discovery, it was observed that the level of circulating NT increased several minutes after a meal, and this increase was more important when the food was enriched with fatty acid (Leeman and Carraway, 1982). Recently, Gui and Carraway (2001) completed this observation by demonstrating that NT acts as a hormone released from intestine following ingestion of fat, and facilitates lipid digestion by stimulating pancreatic secretion. It was also demonstrated that NT enhanced taurocholate absorption from proximal rat small intestine indicating a role in the regulation of enterohepatic circulation. This effect is largely mediated by the release of mast cell mediators, and is regulated by NO (Gui and Carraway, 2004). In conclusion, NT acting as a neurotransmitter or as a hormone, regulates food intake (satiety) and lipids absorption indicating a general role for NT in the regulation of energy balance and in the control of homeostasis.

## NT AND PANCREAS

Immunoreactive-NT (IR-NT) has been detected in plasma extracts and plasma IR-NT elevation occurs in response to nutrient stimuli (Rosell and Rokaeus, 1979). This suggested that NT may influence pancreatic regulation by a hormonal mechanism. IR-NT is present in the pancreas (Fernstrom et al., 1981) and a direct (paracrine) influence on islet hormone secretion was suggested. The presence of NT in the circulation and in the pancreas can be correlated to its effects on insulin and glucagon release (Dolais-Kitabgi et al., 1979) and on the growth of ductal adenocarcinoma of the pancreas (Reubi et al., 1998). Actually, we know that the action of NT on islet secretion is the consequence of the expression of the three NTSRs in normal endocrine pancreas (Beraud-Dufour et al., 2010). By contrast, NTSRs are not expressed in normal exocrine pancreas, their expression being linked with the development of tumors (Kitabgi, 2002; Myers et al., 2009).

Administration of NT increased pancreatic weight, DNA, RNA, and protein contents as well as lipase concentration (Feurle et al., 1987). The proliferative effect of NT on the pancreas has been also demonstrated by Wood et al. (1988). The role of NT and other gastrointestinal hormones like cholecystokinin or gastrin releasing peptide in the growth of normal and neoplastic tissues, including pancreas, has been well documented in a review article (Thomas et al., 2003).

### NT AND PANCREATIC CANCER GROWTH

It is now well established that NT receptors are expressed in exocrine pancreatic tumors and chronic pancreatitis whereas these receptors were not found in normal exocrine pancreatic tissues (Reubi et al., 1998; Wang et al., 2000). Numerous studies have demonstrated that NT stimulates mitogenic signaling pathways and DNA synthesis in human pancreatic cancer cell lines including PANC-1 and MIA PaCa-2 cells (Herzig et al., 1999; Ehlers et al., 2000; Ryder et al., 2001; Guha et al., 2002; Falciani et al., 2010). These growth effects are mediated through NTSR1 stimulation (Iwase et al., 1997). However, a recent study concluded that NT-induced migration of pancreatic ductal adenocarcinoma cells *in vitro* occurs via NTSR3/sortilin pathways (Mijatovic et al., 2007).

### NT AND ENDOCRINE PANCREAS

The first *in vivo* observation that NT displayed a role in glucose homeostasis was performed in the rat where the peptide produced hypoinsulinemia and consequently hyperglycemia (Brown and Vale, 1976). Then after, a more detailed work performed on rat islets of Langerhans demonstrated that NT regulates endocrine pancreatic hormones release (Dolais-Kitabgi et al., 1979). In this study, NT was shown to stimulate insulin and glucagon release at low glucose concentration whereas at high glucose or arginine levels, NT inhibits the release of both peptides. However, in another study performed on isolated neonatal rat islets, NT was unable to alter insulin secretion under high glucose concentration (Fujimoto, 1981). This result may be due to the absence of some NTSRs at this stage of development (Zsurger et al., 1992). The involvement of NT in the regulation of glucose homeostasis has been confirmed in a clinical study dealing with healthy elderly and young subjects. It was demonstrated that in addition to hyperinsulinemia and hypergastrinemia, the postprandial responses for NT were significantly higher in the aged subjects (Arnalich et al., 1990). However, no abnormality in the content of NT was detected in human diabetes, as demonstrated in insulin-dependent diabetic patients and in lean or obese non-insulin-dependent diabetic patients (Service et al., 1986).

## MOLECULAR MECHANISMS OF NT ACTION ON BETA CELLS

### PROTECTION OF BETA CELLS

**FIGURE 1:**
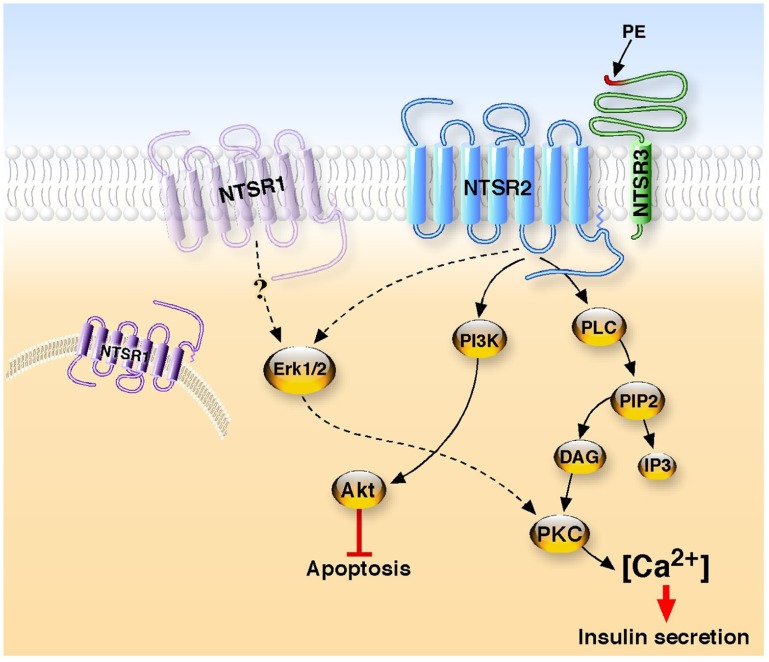
**Neurotensin receptors signaling in beta cells**. Two G protein-coupled receptors, NTSR1 and NTSR2, and one type I receptor, NTSR3, are expressed in beta cells. In one hand, the binding of NT to the complex NTSR2/NTSR3 leads to the stimulation of phospholipase C to enhance the intracellular concentration of calcium responsible for the secretion of insulin. In a second hand, the interaction of NT with the same receptor complex activates the PI3 kinase, resulting in the phosphorylation of Akt to protect beta cells from the apoptosis induced by cytotoxic external agents. Although NTSR1 is expressed in beta cells, an unusual positioning or the absence of the receptor at the plasma membrane may explain the absence of interaction with NT. The propeptide (PE) which is released from the precursor form of NTSR3 displays agonist activity to increase intracellular calcium concentration.

Although there is a lack of evidence for a role of NT in diabetes, there are convincing data for its implication in glucose homeostasis. Surprisingly, no more important studies have been carried out to identify the NT receptor(s) involved in the effects observed. Only very recently our studies investigated the molecular mechanisms involved in the activation of the signaling pathways responsible for NT functions in cultured beta cells. We first demonstrated that all the identified NTSRs are expressed in murine Langerhans islets and in beta cell lines (Coppola et al., 2008; Beraud-Dufour et al., 2009). We demonstrated that NT efficiently protects beta cells from external cytotoxic agents (staurosporine, IL-1beta) through the PI3 kinase pathway (Coppola et al., 2008; **Figure 1**). The NTSR2 partial agonist levocabastine exerts a protective effect similar to that of NT whereas the NTSR1 antagonist SR48692 is unable to block the effect of NT suggesting the predominant involvement of the NTSR2 in the protective action of NT on beta cells. Moreover, we showed that this effect is mediated by NTSR2 via the protein complex formed between the GPCR NTSR2 and the type I receptor NTSR3/sortilin (Beraud-Dufour et al., 2009; **Figure 1**). In this case, the role of NTSR3/sortilin has been postulated to direct NTSR2 to its functional plasma membrane compartment as shown for NTSR1 in HT29 cells (Martin et al., 2002) and for the potassium channel TREK-1 in neurons (Mazella et al., 2010).

The protective action of NT on beta cells is of importance since in diabetes beta cell death is generally the consequence of prolonged hyperglycemia and/or hyperlipidemia. Therefore taking into account that NT is released in the circulation after a meal, in particular after lipid absorption, the neurotensinergic system may save endocrine pancreas as previously demonstrated for glucagon-like peptide 1 (for review Desgraz et al., 2011).

### REGULATION OF INSULIN SECRETION

From experiments carried out on rat beta cell lines, we confirmed the dual action of NT, which is able to increase insulin secretion at low glucose concentration and also to decrease the glucose-induced insulin release (Dolais-Kitabgi et al., 1979; Beraud-Dufour et al., 2010). At the cellular level NT, as well as the NTSR2 selective ligand levocabastine, rapidly and transiently increases the intracellular concentration of calcium in Ins1-E cell line. NT-evoked calcium regulation involves PKC and the translocation of PKCα and PKCε to the plasma membrane. A similar response is obtained with levocabastine, indicating that NTSR2 triggers the effect of NT both on insulin secretion and calcium concentration (**Figure 1**). This result is in total agreement with a study from 1978 in which the hyperglycemia action of NT was shown to be mediated through histamine since blockers of both H1 and H2 histamine receptors were able to inhibit the effect of NT (Nagai and Frohman, 1978). Indeed, it is important to remember that levocabastine, originally developed against H1 histamine receptors (Kitabgi et al., 1987), is a selective ligand of NTSR2 (Chalon et al., 1996; Mazella et al., 1996). Moreover, we demonstrated that the propeptide issued from the maturation of the precursor form of NTSR3/sortilin (Munck Petersen et al., 1999) triggers an increase of intracellular calcium and insulin secretion as observed for NT and levocabastine (Beraud-Dufour et al., 2010). The propeptide acts as an antagonist of NTSR3/sortilin (Martin et al., 2003), however it does not block the NT effect. This suggests that NTSR3/sortilin may be also involved, in combination with NTSR2, in the action of NT. We know that NTSR2 and NTSR3/sortilin form heterodimers in these beta cells (Beraud-Dufour et al., 2009). However, we do not know the exact role of NTSR3 in this complex. NTSR3 may regulate the membrane expression of NTSR2, as reported for NTSR1 (Martin et al., 2002) and the two pore potassium channel TREK-1 (Mazella et al., 2010), and so only NTSR2 selective ligand may activate the receptor complex to trigger the effect. NTSR3/sortilin selective ligands (i.e., the propeptide) may also contribute to the final activation. Further studies carried out on NTSR KO animal models would be crucial for a better understanding of the role of each receptor on insulin secretion.

Interestingly, xenin, a peptide related to NT (Feurle et al., 1992) and co-secreted with glucose-dependent insulinotropic polypeptide (GIP) from intestinal K-cells (Anlauf et al., 2000), regulates glucose homeostasis and potentiates the action of GIP on glucose-mediated insulin release (Taylor et al., 2010; Wice et al., 2010). Xenin enhances GIP-mediated insulin release by a mechanism which does not involve direct action on beta cells. Rather, the effect of xenin appears to be mediated by activating non-ganglionic cholinergic neurons that innervate islets (Wice et al., 2010). The effect of xenin in combination with molecules regulating insulin secretion rates is observed only in humans with normal or impaired glucose tolerance but not with type 2 diabetes (Wice et al., 2012). However, although xenin has been suggested to interact with NTSR1 in guinea pig enteral smooth muscles (Feurle et al., 2002) and in the effect of the peptide in food intake (Kim and Mizuno, 2010), evidence for non-neurotensin receptor-mediated effects of xenin has also been documented in rat intestine (Heuser et al., 2002). In the absence of demonstration that xenin interacts directly with one of the three known NTSRs, the effects of this peptide could be mediated by a system which is, at least partly, independent from the neurotensinergic system for the regulation of insulin secretion.

## CONCLUSION AND FUTURE

From the overall data obtained on pancreatic beta cells and islets, it is clear that although the three NT receptors are expressed, the effects of NT involve both NTSR2 and NTSR3/sortilin but not NTSR1 (**Figure 1**). This is intriguing since the majority of the actions of NT both in the brain and in the periphery involves, at least partly, NTSR1, a receptor which was always shown to be expressed and functional at the plasma membrane of NT target cells. One possible explanation of its lack of implication in beta cells could be that although NTSR1 is detected by PCR and Western blot, the protein is absent or not correctly sorted at the plasma membrane. One of the arguments in favor of this hypothesis is that the binding of iodinated NT measured on membrane homogenate from beta cells is totally displaced by levocabastine (personal observation). Unfortunately, in the absence of efficient antibodies directed against NTSR1 for immunocytochemistry, sub-cellular localization of protein expression appears difficult to be correctly investigated.

**FIGURE 2:**
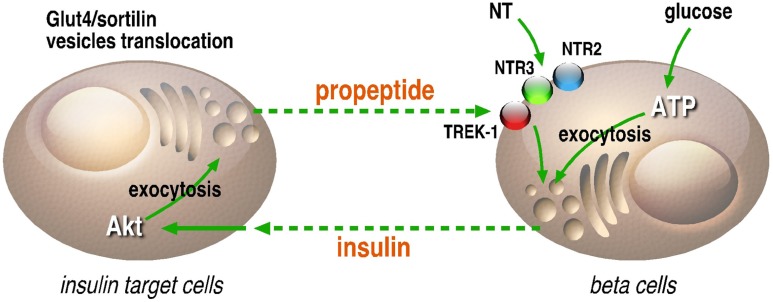
**Regulation loop between insulin secreting and target cells**. In beta cells, insulin can be secreted in the circulation by glucose, NT and also by the propeptide (PE) likely through its interaction with NTSR2 or the potassium channel TREK-1 through NTSR3. In insulin target cells, the activation of insulin receptor by insulin leads to the Akt-dependent translocation of Glut4/NTSR3/sortilin containing vesicles to the plasma membrane where the propeptide can be released in the circulation. This loop of regulation between insulin secreting and insulin target cells is likely involved in fine tuning of glucose homeostasis.

On another hand, the presence of NTSR3/sortilin, as well as the effect of the propeptide on the intracellular concentration of calcium in beta cells, may be also of importance. Indeed, the role of NTSR3/sortilin as a sorting protein and its ability to be translocated to the plasma membrane upon activation by NT (Chabry et al., 1993; Navarro et al., 2001) where the propeptide can be released could serve as a new system of regulation of endocrine hormone release. Moreover, NTSR3/sortilin is also expressed on adipocytes and skeletal muscles, targets of insulin, in which its translocation to the cell surface has been also demonstrated (Lin et al., 1997; Shi and Kandror, 2007). Here again, the propeptide can be released in the circulation and then can regulate by feedback the secretion of insulin by interacting with NTSR3/sortilin and TREK-1 (**Figure 2**). Taken together, these observations refine the regulation scheme of glucose homeostasis through NT and its receptors and will lead to novel research areas with new components and new functions like the propeptide release and action.

## Conflict of Interest Statement

The authors declare that the research was cconducted in the absence of any commercial or financial relationships that could be construed as a potential conflict of interest.
